# “Only your blood can tell the story” – a qualitative research study using semi- structured interviews to explore the hepatitis B related knowledge, perceptions and experiences of remote dwelling Indigenous Australians and their health care providers in northern Australia

**DOI:** 10.1186/1471-2458-14-1233

**Published:** 2014-11-28

**Authors:** Jane Davies, Sarah Bukulatjpi, Suresh Sharma, Joshua Davis, Vanessa Johnston

**Affiliations:** Menzies School of Health Research, Rocklands Drive, Tiwi, Darwin, NT 0811 Australia; Miwatj Health Aboriginal Corporation, East Arnhem, NT Australia; Royal Darwin Hospital, Rocklands Drive, Tiwi, Darwin, NT 0811 Australia

**Keywords:** Hepatitis B, Health literacy, Culture, Portable electronic applications, Language, Indigenous population

## Abstract

**Background:**

Hepatitis B is endemic in the Indigenous communities of the Northern Territory of Australia and significantly contributes to liver-related morbidity and mortality. It is recognised that low health literacy levels, different worldviews and English as a second language all contribute to the difficulties health workers often have in explaining biomedical health concepts, relevant to hepatitis B infection, to patients. The aim of this research project was to explore the knowledge, perceptions and experiences of remote dwelling Indigenous adults and their health care providers relating to hepatitis B infection with a view to using this as the evidence base to develop a culturally appropriate educational tool.

**Methods:**

The impetus for this project came from health clinic staff at a remote community in Arnhem Land in the Northern Territory, in partnership with a visiting specialist liver clinic from the Royal Darwin Hospital. Participants were clinic patients with hepatitis B (n = 12), community members (n = 9) and key informants (n = 13); 25 were Indigenous individuals.

A participatory action research project design was used with purposive sampling to identify participants. Semi-structured interviews were undertaken to explore: current understanding of hepatitis B, desire for knowledge, and perspectives on how people could acquire the information needed. All individuals were offered the use of an interpreter. The data were examined using deductive and inductive thematic analysis.

**Results:**

Low levels of biomedical knowledge about Hepatitis B, negative perceptions of Hepatitis B, communication (particularly language) and culture were the major themes that emerged from the data. Accurate concepts grounded in Indigenous culture such as “only your blood can tell the story” were present but accompanied by a feeling of disempowerment due to perceived lack of “medical” understanding, and informed partnerships between caregiver and patient. Culturally appropriate discussions in a patient’s first language using visual aids were identified as vital to improving communication.

**Conclusions:**

Having an educational tool in Indigenous patient’s first language is crucial in developing treatment partnerships for Indigenous patients with hepatitis B. Using a culturally appropriate worldview as the foundation for development should help to reduce disempowerment and improve health literacy.

## Background

Significant health disparities exist between Indigenous and non-Indigenous Australians resulting in a 10–11 year average reduction in life expectancy for an Indigenous child born between 2005 and 2007 [1]. Liver disease is the third largest contributor (11%) to this gap in life expectancy with chronic hepatitis B (CHB) contributing significantly, in the form of liver cirrhosis and hepatocellular carcinoma (HCC).

CHB is endemic in the Indigenous communities of the Northern Territory (NT) of Australia with prevalence rates estimated to be between 0.8% [for children born in the universal vaccine era (1988 onwards)] and14.2% (for adults born pre universal vaccination) [2–8], this is compared to 1% in Australia as a whole [9]. Despite the availability of effective, government subsidised treatments only an estimated 5% [10] of all people living with CHB in Australia are receiving appropriate management for their infection. This disparity in rates of Hepatitis B and low uptake of treatment is also seen in other Indigenous populations across the world [11, 12].

The barriers to people accessing care for CHB are multifactorial but among Indigenous Australians, include gaps in knowledge, low health literacy and challenges in accessing the appropriate care [13]. Both a recent situational analysis [14] and a qualitative study [15] in the Torres Strait region of Australia identified low levels of knowledge about CHB both in health care providers and Indigenous Australian patients with CHB. Christie et al. [16] have explored views of health literacy in the particular cultural context of remote Indigenous communities in the NT, as well as carrying out a scoping study looking at ways to improve health literacy in this region. Christie (2010) suggests that “*effective health literacy is largely to do with effective communication*” (p.40). Based on their research, they argue that building on an individual’s existing knowledge using a culturally appropriate approach (i.e. a relevant respectful partnership which is mindful of language, worldview, existing knowledge and beliefs) to achieve a shared understanding of the issue at hand is more beneficial than attempts by health practitioners to simply ‘transfer’ biomedical knowledge to their patients [17]. Although many health promotion or information resources exist for hepatitis B [18], all the above [13–17] studies as well as the Australian National Hepatitis B strategy [19] highlight the lack of culturally appropriate resources, in particular visual and multimedia resources, available to facilitate shared understandings of Hepatitis B and strengthen health literacy.

In the context of the NT Indigenous population, English is usually a second (or even third or fourth) language; therefore, achieving effective cross cultural or “culturally safe” communication can be challenging, as has been extensively documented in health care settings over the last decade [20–22]. Miscommunication between health providers and patients has been reported to be pervasive, however using interpreters and translators is perceived to be only part of the solution [20]. Different worldviews and knowledge systems that exist among Indigenous Australians, including alternative concepts of physiology, pathology and disease causation also contribute [23]. An often misinformed assumption by health providers of shared understandings [20], along with the absence of opportunities and resources to construct a body of shared understanding perpetuate this miscommunication. Two specific factors, culture and worldview, are increasingly acknowledged as important antecedents contributing to health literacy [24–27]. There are a myriad of different definitions of culture; when referring to culture in this paper we use the broad definition of the culture of a society as “*… the totality of its shared beliefs, norms, values, rituals, language, history, knowledge and social character”* [28].

Participatory projects working with Indigenous communities in the design and development of health education resources have been successful in improving health literacy and participation in healthcare in other disease areas [29, 30].

The aim of this research project was to explore the knowledge, perceptions and experiences of remote dwelling Indigenous adults and their health care providers relating to hepatitis B infection. We also aimed to gauge interest among Indigenous participants in further knowledge of this disease and gain perspectives on how and in what format people could best acquire the information they needed. This was the first stage of a wider participatory action research (PAR) project with the intention of using the results as the evidence base to inform development of a culturally appropriate Hepatitis B educational resource.

## Methods

This project was undertaken in northern Australia between July 2012 and December 2013. It was based at the health clinic of a remote community in Arnhem Land, 521 km northeast of Darwin (the capital of the NT). This community has a population of 2,124 with an average age of 24 years; 89% are Indigenous Australians and only 9.5% of the population speak English as their first language. There is an average of 4.2 people per available bedroom and 78% of households are considered to be overcrowded. There are three general stores, a school, a library, a health clinic as well as a police station and a community church.

The overall project design was based on PAR principles; specifically, ongoing consultation, reflection and discussion with the community throughout each iterative cycle. JD (a female non-Indigenous researcher and clinician with experience in working in a cross cultural environment) and SB (a female Indigenous researcher and health worker in the remote community) worked alongside each other in constructing the interview schedule, recruitment, data collection, analysis and interpretation. This paper reports the results of the first part of this project which was the first formal PAR cycle and provides the evidence base for the development of a culturally appropriate educational tool for hepatitis B, the second phase of the project (details not presented here). However prior to this project informal discussions regarding the issues facing the community with respect to the burden of disease produced by Hepatitis B, the lack of community understanding and the difficulties health workers have in explaining Hepatitis B to community members had been discussed within clinic meetings and with the visiting liver clinic service. The impetus for the project came from the community clinic. Their enthusiasm for the project led to the development of a collaborative research partnership between the community clinic, the Royal Darwin hospital liver clinic and Menzies School of Health Research and establishment of the formal PAR process.

Ethical approval for the study was obtained from the Human Research Ethics Committee of the Northern Territory Department of Health and Menzies School of Health Research (HREC) as well as Miwatj Health Aboriginal Corporation (an Aboriginal-controlled health service representing communities across East Arnhem Land) and Charles Darwin University.

Semi-structured interviews were carried out with 3 groups of people; key informants (health clinic staff, community health educators, liver clinic staff - both urban and remote, - and doctors and nurses, Indigenous and non-Indigenous), Indigenous people living with CHB and Indigenous community members. Interviews explored the background of the individual, their hepatitis B knowledge, their experience of health communication/education about hepatitis B, available resources and their perspectives about potentially useful educational tools. All participants were shown two existing resources; an animation about the liver and its function (chosen as it was part of an electronic education package targeted at Indigenous Australians) and a flip chart, (developed in Victoria, Australia, intended for use in the clinic setting and aimed mainly at Asian individuals) about hepatitis B and asked to comment on them as a way of generating ideas/preferences for any future educational tool. Patient and community member interviewees were also asked from where they acquired their knowledge about HBV, what influenced their current understanding, and barriers to understanding (Table 1).

**Table 1:** **Interview guides used for semi-structured interviews**

	Key Informants	Hepatitis B patients and community members
**Current role background demographics**	experience with regard to;	Role within family, community, work
	viral hepatitis	Social situation, children, partner
	Indigenous health	Schooling – what age left
	within East Arnhem land	Reason for clinic attendance today
		Hepatitis B status - when first knew about Hepatitis B status
**Hepatitis B knowledge**	own level of knowledge, where did this come from your perception of the general Indigenous populations knowledge	What do you understand by the phrase Hepatitis B infection
	Indigenous patients knowledge	**If no knowledge move directly to communication section**
	what do you think are barriers to increased knowledge (e.g. language, cultural, knowledge systems, health beliefs)	What do you think it is/does, does it concern you
	do you think increased knowledge will make a difference to patient adherence/outcomes, why, why not	How do you think you get Hepatitis B
		Is it a problem for you or your family
		How did you learn about Hepatitis B
		Whose opinion, story do you most trust, why, how did this person talk to you
		What do you think about doctors/nurses/AHWs opinions/beliefs
**Communication**	Are you involved in; testing patients, explaining results, counselling regarding treatment options, follow up, screening protocols	What is your experience of talking to doctors/nurses/midwifes /AHWs about your health in general/specifically Hepatitis B
	If yes to any of above what is your experience of this process with regard to communication, provision of education (is it easy, what do you find difficult about it, do you think it is done well, what do you think are the problems, how can we overcome them) any examples of real life situations with respect to any of these issues	How do you think this could be better
		Do they use interpreters, how does this help you
		Do they use pictures/flipcharts/other tools to help you understand
		Which of these things do you prefer
		Do they help, why
		How would it help you if you understood more about your health/hepatitis B
**Available resources & ideas for educational tool**	Have you used any resources to help with communication/health education, if so details	What kind of thing do you think would help you to understand better – flip chart/pictures/talking/electronic/tablet/phone based tool
	Do you use/need/have available interpreters	Look at these resources, are any of them attractive to you, which do you like, what do they say to you
	Do you have an idea of what kind or resources might help	What are your thoughts about an ipad based resource
	Please look at this collection of resources/images and tell me what you think about them, would they be helpful in this context, how could we use them in this context	What things do you want to know about
	How do you feel about an electronic/tablet based resource, how would you see that working, advantages vs disadvantages	What about pictures
	Are there any resources you like from different situations	What about language
	What format do you think will work (electronic, flipchart, other)	What about interaction
	Any ideas about what should be included	Will it help, why, why not
	What about language, images, interactive or not	

Key informants = health clinic staff, community health educators, liver clinic staff both nurses and doctors, Community members and Hepatitis B patients = Indigenous people living in the remote community with and without Hepatitis B.

JD and SB recruited participants into the study and carried out the interviews; both had received specific training in interview techniques prior to the commencement of the project. All patients were given the option of an accredited interpreter in their first language if this was not English both for the process of obtaining written informed consent and the interview itself.

A mixture of purposive (non-probability sampling in which the researchers suggest who to approach to be included in the study based on them possessing certain characteristics [31]) and network (using existing participants to suggest other people to approach [32]) sampling was used to recruit individuals from a range of different backgrounds with a proportionate mix of gender, age and hepatitis B status. The majority of participants were recruited through the community clinic and the hospital liver clinic; however some individuals were recruited through the social and professional networks of the research team.

Interviews were carried out in numerous settings ranging from the community clinic, our hospital clinic, our research institution, individuals’ homes and gardens, under trees and at an international conference (8th Australasian Viral Hepatitis Conference, Auckland, September 2012). Interviews were audio recorded and ranged in duration from 20 to 45 minutes. Information collected in Yolŋu matha was translated into English in real time by the accredited interpreter and meaning and understanding clarified by SB (bilingual researcher present at all interviews carried out in Yolŋu matha) as part of the recording. Transcription of the interviews was in English.

An audio diary of the real time experience and reflections on the interviews was kept by JD and SB and included in the data analysis. All participants were offered an AUD$30 electricity voucher in recognition of their time and effort in contributing to the study.

**Figure 1:**
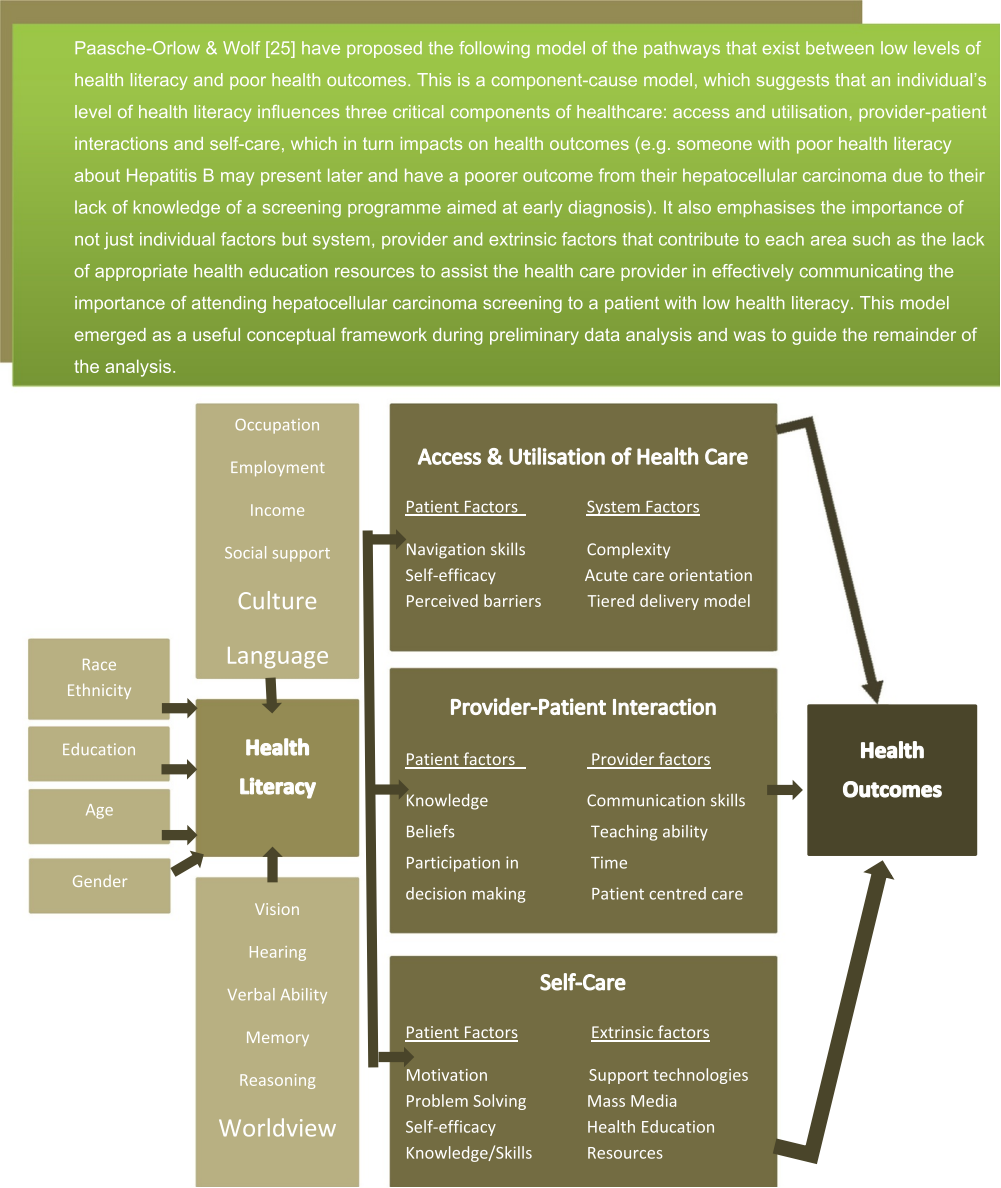
**Adapted version of Paasche-Orlow & Wolf’s model of the pathways linking health literacy and health outcomes** [25] **.**

In the process of exploring patients’ and providers’ knowledge, experiences and perceptions of HBV, data emerged on the potential impact of low levels of health literacy on healthcare interactions and therefore future health outcomes as well as the pathways through which this may occur. As such, we have used Paasche-Orlow & Wolf’s model [25] Figure 1 of the pathways that exist between low levels of health literacy and poor health outcomes as an organising model for our data analysis.

Data analysis was carried out by JD and SB, with input from VJ and JSD. It commenced with the first interview and was continuous throughout the project. Data immersion consisted of carrying out the interviews, reading the transcripts and listening to the audio recordings multiple times on multiple occasions dispersed over time. Sections of text were organised into codes based both on the categories covered in the interview schedules and also inductively as the text was digested and understood. Codes were also reflected upon with reference to the Passche-Orlow & Wolf model in particular with regard to the similarities and differences in using this model in this particular cultural context (Yolŋu people) for this particular disease (Hepatitis B). Concurrently and inductively the codes were organised into broader categories and themes. On multiple occasions clarification was sought regarding the cultural context of specific terms and ideas from SB. SB returned to individual participants to verbally clarify findings on a number of occasions however transcripts were not routinely returned to participants for checking. Data were organised and managed in NVivo 10 (QSR International Pty Ltd, Victoria, Australia).

JD, SB, SS & JSD are all clinical care providers as well as researchers and acutely aware of the ethical implications of this within this project particularly for those individuals interviewed who were Hepatitis B patients. Careful explanation of the fact that the research project and an individual’s clinical care are completely separate and mutually exclusive was undertaken with the Hepatitis B patient group in particular. Care was taken to conduct the interviews completely separately in both time and location from any clinical care so as to maintain this separation.

We adhered to the RATS guidelines in reporting this project.

## Results

Thirty two semi-structured interviews were carried out between July and September 2012. Participants consisted of clinic patients with hepatitis B (11), other community members (9) and key informants (12). Twenty-four (75%) were Indigenous people. Median age of participants was 45 years (IQR 35–55) and 18 (56%) were female. Highest level of education attained was junior school for one individual (3%), secondary school for 23 (72%) and tertiary education for 8 (25%). All participants had the opportunity to use an interpreter; 17 interviews were carried out using a Yolŋu matha interpreter (the principal Indigenous language spoken in the community). The remainder were carried out in English.

### Knowledge about hepatitis B: “Only your blood can tell the true story”

There was a distinct lack of biomedical knowledge regarding CHB, especially in the people living with CHB group, and even among those who had been previously reviewed in the liver clinic and/or were currently on oral antiviral treatment for CHB. People living with CHB and community members generally acknowledged that they did not know or have any understanding of what hepatitis B was and were commonly unable to attempt any explanation on direct questioning.

However, when contextual translation was provided in Yolŋu matha some understanding often emerged:

*“Something like that person will get that virus inside the body. Sometimes he [the virus] will be gone and sometimes will stay there for bit long. That’s the story I know”.*

*“When I see people with hepatitis they have a yellowish thing - eye - you know just around the eye balls and that thing to me, it tells me that the person either have a hepatitis or kidney failure”.*

*Indigenous community member*

The word “germ” and an understanding of germs being micro-organisms that required a microscope to visualise them was recurrently touched upon, with specific reference to previous education programmes and research projects carried out in the community both by The Aboriginal Resource Development Service (ARDS) in Darwin and Menzies School of Health Research. These experiences appeared to have led to an increased understanding of biomedical concepts around infectious diseases in general and were discussed in a positive light.

Despite this many misconceptions about hepatitis B from a biomedical perspective were identified, particularly around causation and transmission. In particular the ideas that CHB can be caused by smoking, lifestyle factors, diet and lack of exercise were frequently reported by community members:

*“Maybe because I was washing myself too much in cold water it may have caused the sickness or me sleeping outside”.*

*“When you smoke you get the sickness in the lungs and in the liver”.*

*Indigenous Hepatitis B patient*

This was also reflected in comments made by numerous people that before “western influences” CHB didn’t exist as a problem; it was a “new” sickness that people did not really know much about and could be prevented by reverting to a more traditional lifestyle.

Many people reported that their underlying beliefs about health and disease are based on traditional medicine including sorcery as causation of disease and traditional plant-based remedies as treatments. Although there were no bush medicines reported that can be used to specifically treat CHB, a remedy made from paper bark trees was described as being used and felt to be effective for liver sickness in general. The biomedical or “balanda” (white person) version of hepatitis B was very much seen as an alternative explanation; new information that didn’t exist in previous generations.

There was also some confusion surrounding Human Immunodeficiency Virus (HIV) and CHB. Some community members reported that the two diseases were one and the same sickness. This misunderstanding appeared to contribute significantly to the sense of stigma or shame around a diagnosis of CHB, and that it had to be kept a secret because of what it might reveal about sexual orientation or partner preference. As well as this, the opinion that an individual patient may be to blame in some way for acquiring CHB, which appeared to be centred on awareness that CHB could be sexually acquired, was recurrently voiced.

This lack of biomedical knowledge was not confined to the patient and community members. Some key informants, both Indigenous and non-Indigenous, also acknowledged that they found it a difficult area to understand clearly themselves. Multiple health professionals reflected on the role of working in an endemic setting seeing a high volume of people living with CHB as necessary to achieve true competency in the management of CHB, stating that prior to this, their understanding was more superficial.

The topic of hepatitis B is part of the routine curriculum studies undertaken by Aboriginal health workers (AHW) and this appeared to be the origin of knowledge for this group, as similar concepts and responses were reported. The concepts of mother to child transmission, sexual acquisition and the infectiveness of blood and other body fluids were expressed by several AHWs; however they were less clear about the natural history of the disease, the interpretation or meaning of blood test results, and the potential for treatment or intervention.

### Perceptions of hepatitis B: “It’s like a silent killer; I can drop dead anywhere so I take my tablets and pray”

People living with CHB and community member perceptions about CHB tended to portray the disease in a negative light, describing it as a “*scary sickness*”, a *“serious infection*”, a “*big sickness*”. People living with CHB in particular described fear as a motivating factor for their actions and behaviours, which either pushed them to take their tablets to prevent imminent death or made them too afraid to attend the clinic, so acting as a barrier to receiving any care.

Within the key informant group there was recurrent reference to the many more urgent competing health priorities in remote communities, such as ischemic heart disease, diabetes and renal disease. CHB, owing to its long term, insidious or asymptomatic nature, in combination with the lack of appropriate resources, resulted in it being neglected and often not adequately addressed. Multiple logistical issues were also felt to contribute to an almost fatalistic view of what was achievable, such as: the remote and dispersed nature of the patient population; the difficulty accessing secondary care physicians and investigations, especially liver ultrasound; the turnover of health care professionals, and lack of continuity of care. In the context of these factors it was perceived that CHB is just too complex a problem to tackle. It was also noted that even where good quality educational resources are available for other diseases, they are rarely used in clinical practice. Instead, they sit on a shelf gathering dust or the technology to use them is either not there or does not work. It is not clear if this is because they are not useful, did not have community input into their development or have not been well implemented or evaluated.

*“People (with Hepatitis B) tend to be asymptomatic for long periods in contrast to chronic diseases like ischemic heart disease, chronic airways disease, chronic kidney disease, diabetes, and day to day problems that people can identify as being directly linked to the condition so it tends to be way down the list of priorities”.*

*Non-Indigenous health worker*

*“The system relies on people being involved for the long haul and yet there’s not a single clinic where we were outlasted by the clinic or the nurse manager of the clinic or the GP where we were there for longer than anyone else in all of the East Arnhem Clinics”.*

*Non-Indigenous health worker*

*“I think, I mean working in the top end I’ve seen a lot of really nice materials that have been developed educationally and flip books and things. In my experience they’re rarely used”.*

*Non-Indigenous health worker.*

Among non-Indigenous key informants there was a perception that it was not possible to translate certain key words such as ‘liver’ and ‘kidney’ accurately into Yolŋu matha and hence adequate explanations of hepatitis B were challenging to achieve even with a translator.

An Indigenous community member working as a translator, however, said that this was not true.

*“Most of the time by and large Yolŋu are hunter gather people. They can cut up a kangaroo, wallabies; they can identify those things [liver and kidney] pretty well, they can make that distinction. It is common knowledge to be able to identify them, there are clear words for them [liver and kidney] and they are different”.*

*Indigenous community member*

### Experiences of living with Hepatitis B: towards a shared understanding

Non-Indigenous individuals in the study (all key informants) tended to significantly overestimate the depth of shared understanding between themselves and Indigenous individuals when discussing CHB. When reviewing existing resources with the non-Indigenous health workers there was recognition that there were too many medical terms and a feeling that they were too detailed in content. However, the general concepts that were explained in these resources were felt to be appropriate. Indigenous participants also described an excess use of jargon but also reported that the concepts used were foreign and difficult to relate to.

*He is saying he’s been to the clinic, they have explained several times. Sometimes he doesn’t understand [what they are saying], especially the doctors.*

*Indigenous Hepatitis B patient*

This lack of shared understanding was also touched upon when discussing the use of AHWs as translators in the context of clinic consultations about CHB. Although a few of the doctors with extensive experience of working in a remote community environment had good insight into the difficulties AHW may face in explaining biomedical concepts, there was a general feeling that having an AHW with them during a consultation to translate their biomedical explanation was adequate to achieve a shared understanding. In stark contrast to this, AHW participants reported finding this expectation overwhelming as they did not feel sufficiently equipped to be able to facilitate a satisfactory explanation due to their lack of understanding of what was being said.

*If I don’t understand the message then how am I gonna convey it.*

*Indigenous health workers (key informants)*

Multiple patients voiced the concern that they were asked to have many blood tests related to their diagnosis of CHB, without receiving adequate explanation of their purpose, and that there was a lack of follow up to receive and discuss the results. This lack of understanding and communication left them feeling worried, angry and frustrated and in several cases like the clinic staff were purposely hiding something from them, resulting in a lived experience of disempowerment and inferiority.

*“I hold my temper at that time, when I don’t get my results back I feel like I need, I want to do something, like smash windows or something here at the hospital”.*

*“I figured there was something wrong with me when they kept on requesting more and more bloods from me”.*

*“That’s one of the things. Sometimes doctors hide something to the patient and they don’t want to tell straight”.*

*Indigenous Hepatitis B patients*

The results described so far highlight factors which all contribute to the patient-provider aspect of the Paasche-Orlow & Wolf model (Figure 1). As well as clearly impacting on an individual’s Hepatitis B specific health literacy these factors appear to shape healthcare interactions, potentially representing a foundation step in the pathways that exist between low levels of health literacy and poor health outcomes in Indigenous Australians.

### The importance of language in health education and healthcare interactions

Indigenous participants across all 3 groups overwhelmingly cited language as the single most important feature of any potential educational resource and also as the most significant barrier to achieving effective cross cultural communication.

*“She’s saying, she doesn’t understand, it’s not much meaningful. The words are big words, the numbers are not good, and the words are not good. Should be in language”.*

*Indigenous Hepatitis B patient*

On multiple occasions through the process of interviewing (at the request of individuals normally in the patient group), we used a trained interpreter to provide a brief clinic style explanation of CHB, and this appeared to be able to significantly increase an individual’s understanding of their illness.

It was however emphasised repeatedly that the translation process was not simply a case of turning the English into Yolŋu matha and that multiple steps were needed; to ensure the individual translating has adequate understanding, to allow/enable contextual translation, to communicate the message via the interpreter in the appropriate language, to check understanding in language, to ask the interpreter to back translate the participant’s understanding and to clarify any miscommunication, as well as great care not to simplify the message too much such that the detail was lost. Indigenous participants perceived that the best path is to remove all medical jargon and acronyms and translate the simple English into Yolŋu matha, using accurate but “*culturally safe”* concepts. The value and preference for visual aids, again of a culturally safe and accurate nature, was a predominant comment.

It became apparent over the duration of the project that there was a lack of shared understanding of the word “silent” between non-Indigenous key informants (health workers) and patients in the context of hepatitis B. Whereas the non-Indigenous health care professional may use the word ‘silent’ to describe the immune tolerance (early stage CHB when viral load is high but minimal liver damage is occurring) or immune control phase (later stage CHB following e antibody seroconversion where viral load is low and minimal liver damage is occurring) of hepatitis B, a Yolŋu patient or AHW may interpret this to mean that the sickness is brought about by sorcery^a^, with negative connotations of retribution or punishment. Although not held by all, this was a commonly held belief voiced amongst the Indigenous people interviewed.

### The relationship between culture and communication in health education

Culturally important relationships between certain individuals, which health care providers may not be aware of, were seen as a barrier to effective communication. For example; a well-respected senior male elder in the community may feel uncomfortable with having a younger female interpreter in a medical consultation, as it would infer something negative about his knowledge of the subject or ability to understand the health care worker and so decline the assistance of an interpreter altogether. This can then result in the individual having an inadequate understanding of the information presented to them.

The importance of gender sensitivity, not only in a clinical scenario but also in any potential educational resource was touched on by individuals in all groups. The ability for people to speak honestly and in detail about hepatitis B was felt to be culturally difficult between individuals of different gender. Patients and community members felt this to be more important if the gender mismatch was between two Indigenous individuals and not as significant if the second individual was a non-Indigenous individual or a health care professional. However some non-Indigenous health care professionals felt that consultations between a health worker and patient of the same gender tended to result in improved cross cultural communication and improved rapport.

### Motivation to understand more about hepatitis B: *“we want to learn more about this sickness”*

Despite a lack of biomedical knowledge, Indigenous participants passionately voiced a desire to understand more about hepatitis B. The importance of telling the full and true story was emphasised, in not missing out the details, but finding a culturally appropriate contextual translation to allow a shared understanding of the important information. Indigenous participants were enthusiastic about spreading this knowledge to all to whom it may be relevant in order to allow them to make choices about seeking management. Both Indigenous people living with CHB and community members perceived that the moral and ethical obligation was on “us”, the health care providers, the ones giving injections (vaccination) and taking blood tests to ensure patients were appropriately informed. This understanding was felt to be very powerful in facilitating autonomy and respect, as well as being vital to a respectful patient – health care professional relationship.

*“She’s saying she wants to learn more about this hepatitis B so she can pass the story to her people, to her family. And to encourage them to come to the clinic and have a check-up”.*

*Indigenous Hepatitis B patient*

### A culturally appropriate education resource: what we need…

When discussing educational resources, non-Indigenous key informants reported that an analogy with hepatitis B using a local animal (e.g. a crocodile or snake) to represent how the virus can lie dormant in the liver and then suddenly attack resulting in serious health consequences would be culturally appropriate. By contrast, Indigenous participants generally preferred more medical imagery requesting to see a real human-like figure with a real liver, and a story based in a culturally appropriate setting. One participant remarked that the majority of local animals are hunted as food by community members, so it would be counterproductive to use them to explain a human sickness - people would then think they could get the disease from the animal.

A strong desire to understand the detail about Hepatitis B was recurrently expressed but the need for contextual translation done in a culturally appropriate way was stressed. In general, Indigenous participants reported a preference for an electronic format with an emphasis on interactive pictures and less text. If text is utilised, it was clear from participants that it must be in Yolŋu matha and spoken as well as written.

There was a recurrent specific request for a separate “women’s business” section to speak about the issues specifically related to pregnancy.

**Figure 2:**
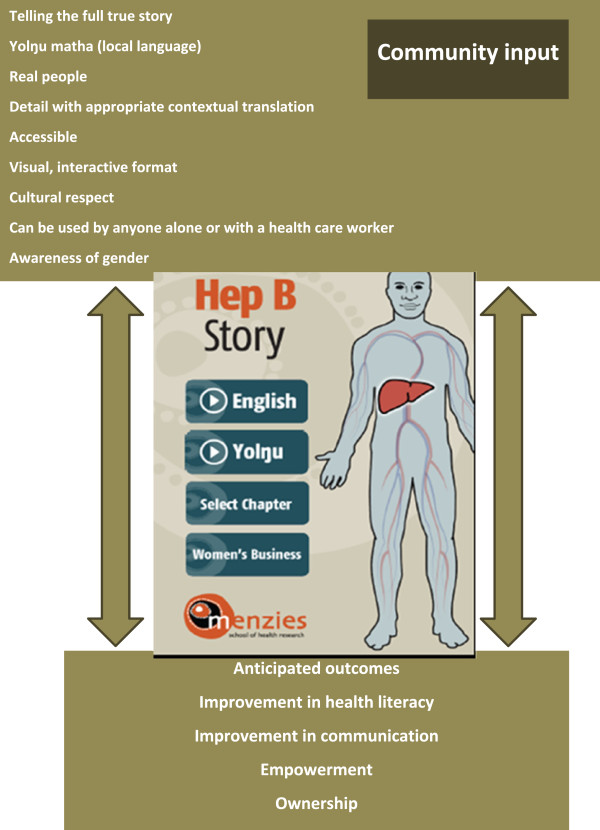
**Key features of the results from the qualitative study that have formed the evidence base for the development of a culturally appropriate tool aid in the development of a shared understanding of the Hep B story with Yolŋu people.**

Figure 2 summarises the important aspects from the results which have been taken forward into the process of developing a culturally appropriate tool to aid in the development of effective treatment partnerships for Indigenous patients with CHB.

**Figure 3:**
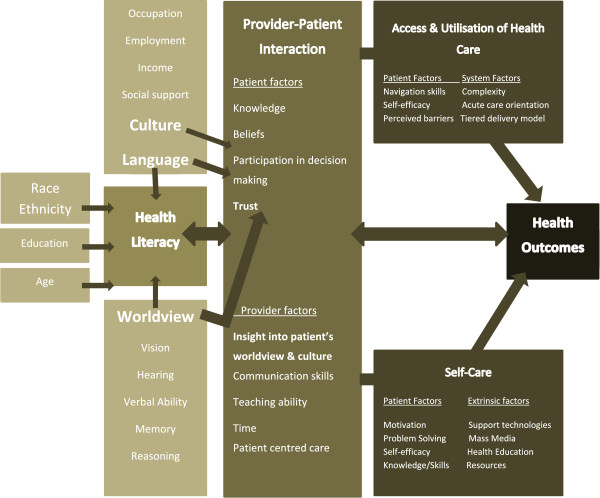
**Paasche-Orlow & Wolf’s model adapted based on the results of this qualitative study to highlight how the relationships between health literacy and poor health outcomes may operate for Indigenous Australians with respect to hepatitis B.**

In light of our results we have adapted Paasche-Orlow & Wolf’s model Figure 3 to highlight how the relationships between health literacy and poor health outcomes may operate for Indigenous Australians with respect to hepatitis B.

## Discussion

This study documents low levels of biomedical knowledge about hepatitis B which appear to be influenced by a multitude of factors including culture, gender, competing health priorities and a lack of shared understanding. Pessimistic almost fatalistic perceptions of the disease predominated across all groups of individuals interviewed. In terms of experiences the major theme identified was communication particularly the importance of having information available in an individual’s first language to aid in effective cross cultural communication. Indigenous individual’s repeatedly expressed a desire for increased knowledge and insight into the ability of this knowledge to reduce disempowerment and improve Hepatitis B specific health literacy. Ideas as to how to best enable this to happen included using visual aids, electronic formats, simple language and the absolute requirement for information to be available in Yolŋu matha.

Knowledge and beliefs are important patient factors in the patient-provider interaction component of Paasche-Orlow & Wolf’s model linking low levels of health literacy and poor health outcomes. A lack of biomedical knowledge about hepatitis B was identified in Indigenous individuals across all groups interviewed. This is consistent with data from Indigenous individuals in the Torres Strait [15] as well as non-Indigenous Australians from culturally and linguistically diverse backgrounds [13]. Lack of knowledge and erroneous beliefs about hepatitis B, as well as contributing to low levels of health literacy, may lead to a reduced ability or willingness to participate in decision making about management plans. This in turn may influence adherence with the plan and subsequent necessary self-care activities. Multiple factors affecting the provider side of the patient-provider interaction were also identified. Communication skills to allow shared understandings to be developed as well as insight into how best to achieve this are crucial in our context, where there are multiple competing priorities; however lack of these skills is identified in our results as an ongoing barrier to achieving shared understandings. In the context of Australian Indigenous peoples where English is not the first language and culture and worldview are very different we would suggest that the patient-provider interaction not only significantly contributes to health literacy but is a pre-requisite to allowing access & utilisation of care and self-care to occur and so ultimately influencing health outcomes (Figure 3).

As well as the patient-provider factors described above, extrinsic factors such as support technologies, health education and resources are identified as key factors to allow optimisation of self-care. The wider project that this research is part of was initiated due to a lack of culturally appropriate resources about CHB for use in clinical practice. Our data identified a real desire for more knowledge and understanding around CHB for all in the community to motivate and empower people living with CHB and community members, which in turn should increase self-management in relation to CHB. Our results identify a clear ambition by community members and people living with CHB towards ‘critical health literacy’ as defined by Nutbeam et al. [33] as the tertiary level of health literacy encompassing not only communication of information and development of personal skills but also personal and community empowerment.

There is now increasing experience with the use of innovative, interactive, internet, mobile phone and tablet-based resources to improve health literacy in other settings [34, 35]. In the context of Indigenous Australia, several groups have produced apps in the area of mental health [36] but robust evaluation of their value is still awaited. In northern Australia, Christie’s research group has proposed a tablet-based, easily transportable, touch pad body resource, which does not contain any embedded health messages, but rather focuses on aspects of a healthy body. Their vision is that this could be used as the foundation for a further discussion about the impact of chronic diseases on the body and how treatments act to return the body to a healthy state [16]. The evidence derived from this project that will be taken forward to phase 2 of the PAR process and used to guide the development of a culturally appropriate educational tool about Hepatitis B is summarised in Figure 2.

Effective communication is not only central to improving health literacy [37], it is a crucial element in achieving culturally safe healthcare, which in essence can be defined as “*shared respect, shared meaning, shared knowledge and experience of learning together”*[38]. More recently, research suggesting that some Indigenous patients believe that health care workers deliberately withhold information from them highlights the extreme lack of trust that can develop as a consequence of ineffective communication [21]. As communication transcends all aspects of health literacy, hence “culturally safe communication” at both a system and individual level is clearly integral to its improvement. Culturally safe communication has also been suggested as being important in reducing ethnic and racial disparities in healthcare [39]. Specifically in the Australian Aboriginal context, involvement of the local community in developing and implementing health education programmes, so they are culturally safe, has been shown to directly influence their effectiveness [29, 30, 40] and attention to worldview and language are argued to be integral to achieving improvements in health education [41]. It is therefore disappointing that more than a decade after the publication of Cass et al’s [20] paper documenting the pervasive nature of miscommunication between Indigenous people and their health care professionals, our results show the major barrier to achieving critical health literacy is still poor cross-cultural communication. Consistent with the view of Vass et al. [41] who suggest “the health literacy of Indigenous Australians can be improved by promoting the oral use of the peoples’ first language in the health sphere” Indigenous participants anticipate they will better understand and be able to process and act on information given to them in their own language.

Our results also provide further insight into the complexity of achieving effective and culturally safe communication in this setting, when, for example, the lack of a shared understanding of one word – “silent” – which is used so commonly in clinical practice with hepatitis B patients can lead to such significant misunderstanding. We have also highlighted the potential for miscommunication to be perpetuated in health settings when communities are not adequately consulted about health education and health promotion resources. The well-meaning but mistaken beliefs among non-Indigenous key informants in this study about the appropriateness of using animal analogies when discussing how hepatitis B affects the liver or the mistaken belief that the lack of a direct translation of a word prohibits meaningful translation of key messages, are two examples from our data.

The negative perceptions and fear of hepatitis B as a disease may originate from the low levels of health literacy documented and contribute to stigma and potential non-disclosure of diagnosis as well as having implications for individual clinical care and the success of public health interventions. This pessimism may have been confounded by the lack of shared understanding and different health beliefs about causation in the context of provider-patient interactions. Additionally, the non-Indigenous key informants in this study perceived that there are multiple logistical barriers and competing priorities to providing effective and appropriate long term care for people living with CHB and felt overwhelmed by the task. This negativity is likely to adversely influence an individual’s access and utilisation of care and so contribute to the relationships between limited health literacy with inequitable health outcomes as per Paasche-Orlow & Wolf’s model.

Our study is limited by the fact it only included one community and because of multiple previous education and research projects in this community in the discipline of infectious disease, it is likely that this community has higher health literacy that most regarding infectious diseases specifically. Cultural practices, traditions and world view may be totally different to other Australian Indigenous peoples; however, our findings about the importance of communications and shared understandings are likely to transcend region and apply to all Indigenous Australians. This view is supported by the similarities between our findings, and those of Preston-Thomas et al., who investigated HBV knowledge in a completely different group of Indigenous Australians – Torres Strait Islanders. Although not directly translatable to other cultures, it is likely that the modified factors highlighted in Figure 3 will be of greater importance to those people living with CHB from culturally and linguistically diverse backgrounds, particularly if they are receiving care in a country where the language of health care is not their own first language.

Although low levels of biomedical knowledge about CHB are clearly a significant barrier and an important influence on health literacy our findings resonate more clearly with Christie et al’s [16] definition of health literacy. In this context, what is really critical to improving health literacy is developing a shared understanding between patients and providers, which hinges on effective communication. If we can use the insight we have gained from this study and work with the people who provided it to develop an educational tool grounded in their culture, in their first language and make it easily accessible, that would be a first step to improving health literacy about CHB. Qualitative research using a participatory approach holds promise of breaking cross-cultural barriers in health communication and health care. We acknowledge that there will also need to be appropriate implementation and evaluation of the resulting resource to ensure its success.

## Conclusions

Biomedical knowledge about Hepatitis B is low in this Indigenous community in the Northern Territory, experiences and perceptions about CHB are in general negative and at times nihilistic. However there is a strong desire for increased knowledge and evidence of increased understandings with contextual translation of information. Patient provider interactions leading to the development of shared understandings between Indigenous people living with CHB and the health care professionals looking after them are the foundation for improving health literacy and so health care outcomes related to CHB. Language and using a culturally appropriate worldview are crucially important in developing an educational resource to aid in developing treatment partnerships for Indigenous patients with CHB. Maintaining a participatory approach to development should help to reduce disempowerment and overcome some of the barriers to its implementation and success.

## Endnote

^a^Sorcery as a cause of disease is a commonly held belief in Indigenous communities in Arnhem Land particularly where a death is sudden, unexplained or happens to someone who is seen outwardly as healthy. It can be a form of retribution or punishment but is not always viewed in this way.

## Abbreviations

ARDS: Aboriginal Resource Development Service
CHB: Chronic Hepatitis B
HCC: Hepatocellular Carcinoma
PAR: Participatory Action Research
NT: Northern Territory.
